# Assessment of Contributing Risk Factors Leading to Heart Failure in Patients Who Are Methamphetamine Users

**DOI:** 10.7759/cureus.76479

**Published:** 2024-12-27

**Authors:** Magnus To, Javad Savoj, Joseph Kraft, Joseph Chang, Napatkamon Ayutyanont, Christian Vandeveer, Rajesh Gulati, Mimi Biswas

**Affiliations:** 1 Cardiology, Riverside Community Hospital, Riverside, USA; 2 Internal Medicine, Riverside Community Hospital, Riverside, USA; 3 Cardiology, Texas Tech University Health Sciences Center El Paso, Riverside, USA; 4 Clinical Research, Southern Hills Hospital and Medical Center, Las Vegas, USA; 5 Statistics, Riverside Community Hospital, Riverside, USA

**Keywords:** cardiomyopathy, heart failure, heart failure with preserved ejection fraction (hfpef), heart failure with reduced ejection fraction, methamphetamine

## Abstract

Background: Methamphetamine abuse is a public health problem across the world, and the cardiovascular system experiences a significant effect on the myocardium over time. Methamphetamine is a common cause of heart failure with preserved ejection fraction (HFpEF) and heart failure with reduced ejection fraction (HFrEF). The prevalence and risk factors for HFpEF and HFrEF in this patient population remain unclear.

Methods: This retrospective case-control study is based on a chart review of patients from 139 Hospital Corporation of America (HCA) community hospitals across the United States from January 2013 to December 2017. Active methamphetamine users were identified based on International Classification of Diseases (ICD) codes and a positive urine drug screen during hospitalization. The total number of patients was then divided into two groups: heart failure (HF) vs. no HF. Then exclusion criteria were applied to remove any possible characteristics that might confound the data. The number of patients left was then analyzed.

Results: This study identified 9,518 active methamphetamine users, with 403 patients (4.23%) having HF, 41 having unspecified HF, and 51 having HFpEF only, vs. 311 having HFrEF only, compared to 9,115 in the control group without HF. After exclusion criteria were applied, the HF group had 166 patients, and the control group had 398 patients. Heart failure was significantly higher in men, 65.7% men compared to 34.3% women (adjusted odds ratio (OR): 1.894, p = 0.022). The average ages of HF group patients before exclusion criteria, after exclusion criteria, and in the control group were 52.5 years, 46.5 years, and 42.7 years, respectively. The age difference between the HF group (average 46.5 years old) and the control group (average 42.7 years old) was not statistically significant (adjusted OR: 1.007, p = 0.540). However, the average age of the HFpEF population significantly differs from the HFrEF population, 70.2 years old compared to 47.1 years old, respectively (adjusted OR: 1.053, p = 0.001). Female patients appeared to be more likely to have HFpEF (70.6%) compared to HFrEF (18.9%, adjusted OR: 3.738, p < 0.001).

Conclusion: Amongst methamphetamine users who develop HF, men appeared to be affected more compared to women. This difference may stem from the differences in activities between men and women. On the other hand, age appeared to not play a role when viewing the entire HF group as a whole. However, when broken down into reduced and preserved groups, age appeared to play a significant role in the type of HF that methamphetamine users may develop. The HFpEF age in methamphetamine users appeared to follow the trend of the general population age with HFpEF. The HFrEF rate was about six times higher among methamphetamine users, excluding the population with unspecified HF. Higher rates of methamphetamine abuse are prompting further studies and programs to reduce cardiac morbidity and mortality in this group and decrease the burden on health systems.

## Introduction

Methamphetamine abuse has become a ballooning public health problem, and understanding its effects on the cardiovascular system is an important priority. In 2021, the National Institute on Drug Abuse reported about 2.5 million active methamphetamine users, with mortality rates as high as 32,000 from methamphetamine overdoses [1]. A cross-sectional study of hospital discharge data showed a 270% increase in hospitalizations from 2008 to 2015. Patients admitted for methamphetamine use have consistently worse morbidity compared to patients admitted for other reasons, including increased hospital stay (5.9 vs. 4.7, p<0.001) and mortality (24.1 per 100,000 vs. 21.9 per 100,000, p<0.001) [2].

The significant morbidity associated with methamphetamine use is likely mediated by its effects on multiple body systems, including the psychiatric, neurologic, gastrointestinal, and cardiovascular systems. In particular, methamphetamine use has been shown to affect the cardiovascular system, causing pulmonary artery hypertension and cardiomyopathy [3]. An Australian case series of 371 cases of methamphetamine-related deaths showed that 54% had some cardiovascular pathology [4]. Yeo et al. first demonstrated via a case-control study of patients ≤45 years old that methamphetamine use was associated with a 3.7-fold increase in odds ratio (OR) (1.8-7.8, 95% confidence interval (CI)) for heart failure with reduced ejection fraction (HFrEF)[5]. In fact, cardiomyopathy associated with methamphetamine use appeared to be a distinct and more severe entity, as it was associated with a lower ejection fraction than non-methamphetamine cardiomyopathy (26% vs. 35%, p = 0.009) [4].

The phenotype of heart failure (HF) in methamphetamine abuse is diverse, but the underlying pathophysiology still remains unclear. A proposed mechanism is acute myocardial ischemia through coronary vasospasm, with some studies reporting the development of coronary atherosclerosis, direct myocardial injury from increased p53 activity causing apoptosis, enhanced free radical production resulting in oxidative stress, excessive catecholamines, and altered mitochondrial function [6-9]. Amphetamines can result in dilated, hypertrophic, or stress-induced cardiomyopathy. Dilated cardiomyopathy is believed to result from the direct toxicity of amphetamine to myocytes, whereas hypertrophic cardiomyopathy stems from profound hypertension due to adrenergic stimulation. Stress cardiomyopathy may be a consequence of the physiologic strain from excessive catecholamine release [4].

Identifying unique risk factors and complications of methamphetamine-associated cardiomyopathy (MA-CM) may facilitate a deeper understanding of the disease process. When comparing hospitalized patients with amphetamine use to general hospitalized patients, amphetamine users were more likely to be younger (<65 years old), male, and residing in the Western part of the United States [2]. A retrospective study of 702 patients with a history of methamphetamine abuse found that patients with MA-CM showed a predominance of males compared to controls (86% vs. 58%, p<0.001). Moreover, MA-CM was associated with concurrent alcohol use and hypertension [3].

In this retrospective case-control study, we sought to characterize a population of methamphetamine users in a population of patients presenting to a network of community hospitals. Methamphetamine users who developed HF were compared to patients without HF to identify underlying demographic and clinical factors associated with cardiomyopathy. Elucidating the risk factors may also provide greater insights into the underlying pathophysiology of the devastating cardiovascular complications of amphetamine use.

## Materials and methods

The study was approved by the Hospital Corporation of America's (HCA) Healthcare Graduate Medical Education Institutional Review Boards. Data were extracted from medical records of all patients from 139 hospitals in 20 different states across the United States based on the International Classification of Diseases (ICD) codes from January 2013 to December 2017. All patients with a history of methamphetamine use (ICD-10 code F15 or ICD-9 code 304.4.) and/or a positive urine toxicology for amphetamines on admission were identified. Among patients with a history of methamphetamine use, all patients with a history of HF (ICD-10 code I50 or ICD-9 code 428) were identified.

For the primary hypothesis testing, patients were divided into two study groups: patients with HF and patients with no history of HF. Patients with a history of coronary artery disease (CAD) (ICD-10 codes I20-I25 or ICD-9 codes 413, 410, 423, 429, 411, 412, and 414) were excluded. Remaining HF patients were divided into two groups: those with heart failure with preserved ejection fraction (HFpEF) (ICD-10 I50.3 or ICD-9 428.30) and HFrEF (ICD-10 I50.2 or ICD-9 428.22), while patients with unspecified HF diagnoses were excluded. Patients with HFrEF and HFpEF were diagnosed either with a transthoracic echocardiogram or a left ventriculogram. All patients who had an ejection fraction of <50% were included in the HFrEF group. A distinction between heart failure with mid-range ejection fraction (HFmrEF) and HFrEF was not made. Patients with HFpEF were evaluated by transthoracic echocardiogram and coronary angiogram using several parameters, including pulmonary artery systolic pressure and early mitral inflow velocity/mitral annular early diastolic velocity ratio.

Any patient with HFrEF or HFpEF who had CAD found either by cardiac computed tomographic angiography, nuclear stress test, or coronary angiogram was excluded from the study due to possible ischemic cardiomyopathy that could confound the data. The ejection fraction was evaluated either by a transthoracic echocardiogram or a left ventriculogram. Additionally, other exclusionary criteria were used, including age <18 years, missing labs including brain natriuretic peptide (BNP) and troponins, any prior cardiac surgery, cardiomyopathy from infiltrative diseases, muscular dystrophies, congenital cardiomyopathy, constrictive pericarditis, and active malignancy or suspected malignancy as listed in Table 1.

**Table 1:** Exclusion criteria used to exclude patients with characteristics that could potentially confound the data.

Exclusion criteria
Age < 18
Coronary artery disease
Prior cardiac surgery
Cardiomyopathy from infiltrative disease
Muscular dystrophies
Congenital cardiomyopathy
Constrictive pericarditis
Hypertrophic cardiomyopathy
Restrictive cardiomyopathy
Active malignancy
Suspected malignancy
Missing labs including brain natriuretic peptide (BNP) and troponins

Demographics and cardiovascular risk factors were determined from medical history and compared between different groups. Patients within the young adult age group up to the elderly in their 80s were evaluated. Patients' sex was compared between the different groups. Additionally, other substance abuse, including tobacco smoking, alcohol, and other illicit or recreational drugs, was not thoroughly evaluated. Unfortunately, race and ethnicity were not evaluated in this study.

Odds ratios were used as the measure of association and were calculated using unconditional logistic regression. Normal theory approximation was used to determine the 95% CI for OR estimates. The significance testing of regression coefficients was based on Wald’s statistic. Univariate factors associated with cardiomyopathy at the alpha (α) level of 0.05 of statistical significance were included in the multivariable logistic regression model. The Fisher’s exact test was used for the comparison of categorical variables and the Student's t-test for continuous variables. A p-value of <0.05 was considered to be statistically significant. Statistical analysis was performed using SAS software (SAS Inc., Cary, NC).

## Results

Within this study, 9,518 active methamphetamine users were identified from 139 hospitals located across 20 different states. Among those, 403 patients (4.23%) had a history of HF (51 HFpEF vs. 311 HFrEF vs. 41 unspecified, p<0.01), and 9,115 patients without a history of HF were assigned to the control group. The characteristics of the patients included in this study were evaluated and compared. Table 2 provides details of the patients' characteristics evaluated.

**Table 2:** Characteristics of the patients included in the study CAD: coronary artery disease. HFrEF: heart failure with reduced ejection fraction, defined in this study as ejection fraction less than 50%; LVEF: left ventricular ejection fraction; HFpEF: heart failure with preserved ejection fraction; HF: heart failure; BMI: body-mass index, which is the weight in kilograms divided by the square of the height in meters; BNP: brain natriuretic peptide used as a marker for heart failure. Troponin in this study is not the high sensitivity troponin with a normal range between 0 and 0.04.

Characteristics	N = 9518	Standard deviation
Sex		
Male (%)	6710 (70.5)	
Female (%)	2808 (29.5)	
Smoker (%)	7471 (78.5)	
Non-smoker (%)	2047 (21.5)	
Hypertension (%)	6339 (66.6)	
CAD	8594 (90.3)	
HFrEF, LVEF < 50% (%)	311 (3.3)	
HFpEF (%)	51 (0.53)	
Unspecified HF (%))	41 (0.43)	
No HF (%)	9115 (95.8)	
Average age (years)	46.1	
Age in the HF group (years)	52.5	
Age in the no HF group (years)	42.7	
Age in the HF group after exclusion (years)	46.5	
Age in the no HF group after exclusion (years)	42.7	
Average BMI (kg/m^2^)	28	8.1
Average BNP	1334	6949.9
Average troponin	0.066	0.601

The total number of male patients in this study was 6,710, whereas the number of female patients was 2,808. Smokers and non-smokers were 7,471 patients and 2,047 patients, respectively. Hypertension was observed in 6,339 patients. A majority of the patients had underlying CAD, making up 8,594 patients, or about 90.3%. Patients with HFrEF made up 3.3%, or 311 patients, while those with HFpEF made up 0.53%, or 51 patients. A small group of patients had unspecified HF, making up 0.43% of the 41 patients. The majority of methamphetamine users did not observe HF in 9,115 patients.

The average age overall was 46.1 years. When broken down without exclusion (exclusion criteria as mentioned in the Materials & Methods section) in the HF group and the control group, the age was 52.5 years and 42.7 years, respectively. With exclusion, the average age for the HF group and the control group was 46.5 years and 42.7 years, respectively.

**Figure 1:**
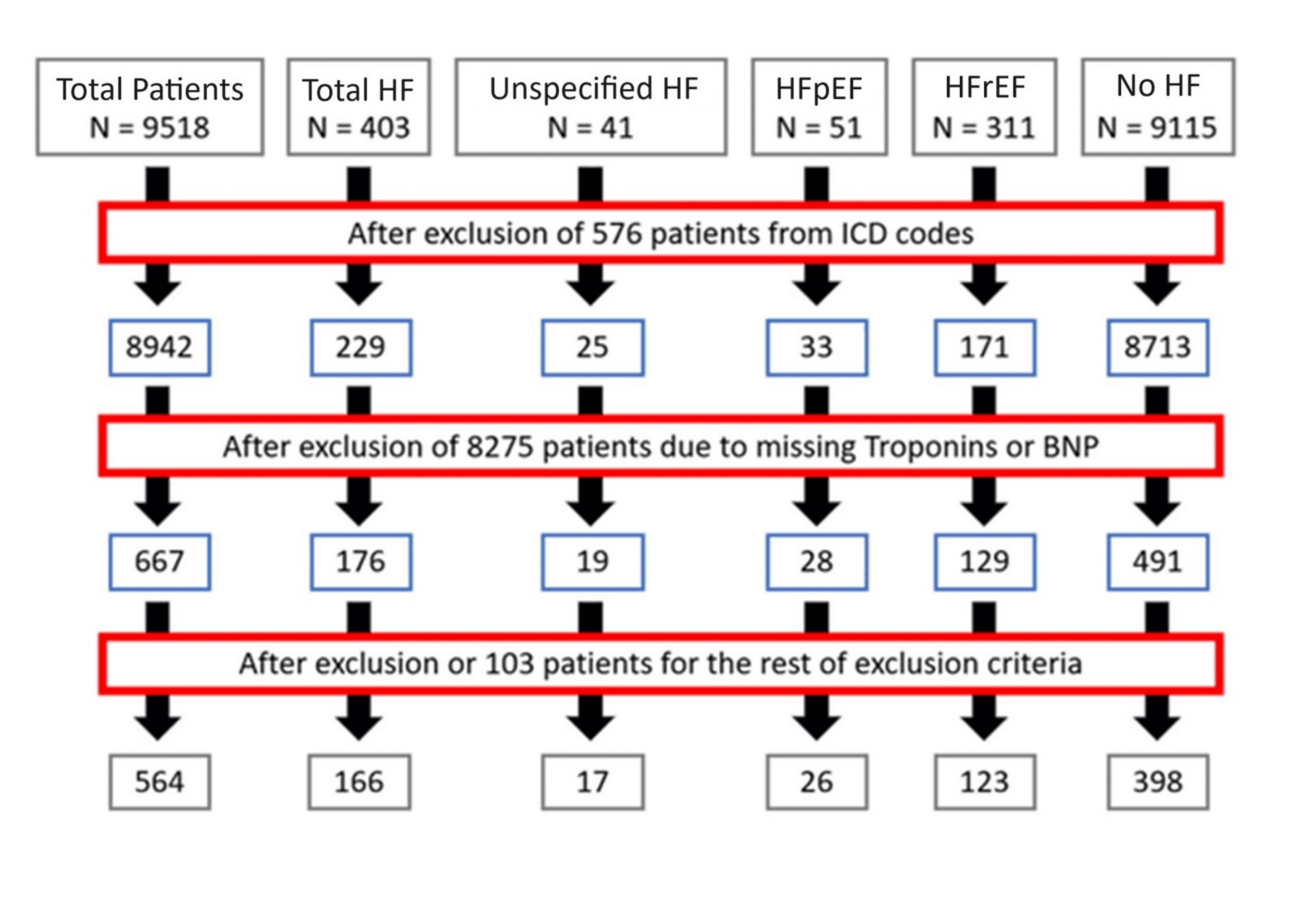
First, the exclusion criteria removed patients based on ICD codes. Second, exclusion based on missing troponin or BNP lab values was done. Then, exclusion from other exclusion criteria was performed. The other exclusion criteria are mentioned in Table 1. HF: heart failure; HFpEF: heart failure with preserved ejection fraction; HFrEF: heart failure with reduced ejection fraction; ICD: International Classification of Diseases; BNP: brain natriuretic peptide

The initial exclusion was from ICD codes, which reduced the total number of patients to 8,942. Subsequently, only patients with information on both troponins and BNP available in their lab reports were included, which further reduced the total number of patients to 667. Afterward, exclusion from the exclusion criteria listed in Table 1 gave the final count of 564 patients. It was observed that 43% of total HF patients had no CAD. In the end, only 166 patients were included in the HF group, as seen in Figure 1. Heart failure was significantly higher in male patients than in female patients (adjusted OR: 1.894, p = 0.022). The average age of the HF group after exclusions was 46.5 years, compared to 42.7 years in the control group. However, it did not show statistical significance (adjusted OR: 1.007, p = 0.54), as depicted in Table 3. There was no significant difference based on a history of smoking or hypertension. Ethnicity was not evaluated between the two groups. Among HF patients, age was significantly higher for HFpEF as compared to HFrEF (adjusted OR: 1.053, p=.001), with average HFpEF and HFrEF ages of 70.2 years and 47.1 years, respectively, as seen in Table 3 [10].

Additionally, female patients were more likely to have HFpEF than HFrEF (adjusted OR: 3.738, p<.001). Out of the total population of patients in the study with non-ischemic HF, either HFpEF or HFrEF, 31.5% of females had HFpEF as compared to only 13.1% of males (Table 3).

**Table 3:** Study comparison of heart failure cases compared to control cases: the comparison of average age between the two groups showed no statistical significance, while the average age of HFpEF vs. HFrEF showed a significant difference and reached statistical significance. HFpEF also appeared to be significant in women compared to men, with a great portion of women having HFpEF compared to men having HFpEF. HF: heart failure; HFpEF: heart failure with preserved ejection fraction; HFrEF: heart failure with reduced ejection fraction; OR: odds ratio

	Total HF	HFpEF	HFrEF	Control	Adjusted OR	P-value
Without exclusion (N)	403	51	311	9115		
Age (years)	52.5			42.7		
With exclusion (N)	166	26	123	398		
Age (years)	46.5			42.7	1.007	0.54
Age (years)		70.2	47.1		1.053	0.001
Female patients HF (%)		18 (70.6)	23 (18.9)		3.738	<0.001
	Female	Male				
Total HF with exclusion	57	109			1.894	0.022
HFpEF with exclusion (%)	18 (31.5)	8 (13.1)				

## Discussion

Methamphetamine use has become an increasingly recognized national epidemic, with mortality from psychostimulants increasing by about 250% between 2008 and 2015 [2]. Our study highlighted the role of cardiovascular disease in this epidemic. It adds to the growing base of knowledge about methamphetamine-associated non-ischemic cardiomyopathy (MA-NICM) patients. While the overall prevalence of congestive heart failure in adults is 1%-2% worldwide and 2.5% in the United States [11], the prevalence of MA-CM was found to be 4.2% in our study, from 2013 to 2017. This is consistent with previous studies showing the rising rate of MA-CM, rising from 1.8% in 2009 to 5.58% in 2014 [12].

Our study in particular showed a rate of NICM of 43% within the broader MA-CM population, similar to the general rate of NICM of 40.8% in the general population seen in previous studies [13]. However, MA-NICM was found to present somewhat differently. In one national registry with 156,013 hospitalized HF patients, NICM was primarily a disease of older patients, presenting on average at 72 years of age [14]. However, our study showed that MA-NICM tends to develop in younger patients, with a mean age of 52.5 years. This is in line with the mean age of MA-CM in other published registries [12].

It is unclear what risk factors influence the accelerated development of cardiomyopathy in young methamphetamine users. The National Health and Nutrition Examination Survey (NHANES) study of heart failure demonstrated that the population-attributable risks of HF were 62% CAD, 17% cigarettes, and 10% hypertension [15]. Since CAD was excluded from our study population, underlying CAD differences could not explain differences in congestive heart failure (CHF) risk. Similarly, our study found no significant differences in hypertension or cigarette use between methamphetamine users and control patients. Mirroring previous studies in the MA-CM population, we found that male sex was a risk factor for MA-NICM [12].

Men appear to be more susceptible in general to methamphetamine-associated toxicity, with significantly higher rates of methamphetamine-related deaths reported in the emergency department [16]. There are many potential explanations for the difference in susceptibility between men and women. Men have been shown to exhibit certain problematic behaviors, such as the use of other drugs, such as alcohol [16]. Concomitant substance abuse has been shown to increase susceptibility to MA-CM, possibly through its enhanced hemodynamic effect and worsening hypertension and tachycardia [3]. In addition, estrogen may confer greater protection to women against methamphetamine-induced structural and cellular toxicity. In a mouse model of MA-CM, male mice were found to have high rates of dilated cardiomyopathy, associated with higher rates of fibrosis and molecular anti-inflammatory markers [17]. This appears consistent with findings in MA-CM patients, who show significantly higher rates of fibrosis and inflammation on endomyocardial biopsy [18].

We also found the MA-NICM population itself to be heterogeneous. Patients with methamphetamine-associated HFpEF were significantly older than their counterparts with HFrEF. On the one hand, this mirrors the general trend. The HFpEF patients are generally older; the average age of HFpEF patients was 74.4 years, compared to 71.7 years for HFrEF [19]. However, our study showed a more pronounced difference in MA-NICM patients. Among methamphetamine users, the average age of HFpEF was 70.2 years vs. 47.1 years in HFrEF. The epidemic of MA-NICM among young patients appears to be driven mainly by an increased rate of HFrEF at a younger age.

In addition to its cost on individual lives, methamphetamine imposes a severe cost on society. The cost of HF was projected to increase from $29.2 billion in 2015 to $63.6 billion by 2035 [20]. Since MA-CM makes up roughly 4.2% of HF patients, it may result in a similar percentage of the overall cost and burden to society. In fact, MA-CM patients may disproportionately affect healthcare costs, given their increased length of stay and worse in-hospital mortality compared to the general population. Moreover, the development of HF also results in indirect costs due to morbidity and mortality. This value, calculated as the cost of lost productivity at home and work, was estimated to be $11.3 billion in 2014 [20]. This may again underestimate the total burden, as patients with MA-CM are younger than the average CHF patient and tend to have worse subjective criteria, such as New York Heart Association (NYHA) class III/IV heart failure [3].

It is therefore important to consider routes to address and potentially reverse the cardiovascular complications of MA-CM. Current therapy involves guideline-directed medical therapy, including angiotensin-converting enzyme inhibitors/angiotensin receptor blockers, beta-blockers, mineralocorticoid receptor antagonists, and sodium-glucose transport protein-2 inhibitors, as well as wearable or implanted cardioverter-defibrillators. Special considerations for MA-CM include medication compliance and persistent substance use. Previous studies have shown that methamphetamine users with human immunodeficiency virus (HIV) have poor compliance with anti-retroviral medication [21]. In addition, two retrospective cohort studies have shown that MA-CM patients who abstain from methamphetamine show higher rates of improvements in ejection fraction and NYHA heart failure class [12, 18]. Cessation is therefore important to improve compliance and potentially reverse the functional and clinical impacts of heart failure. It will be crucial to include substance cessation techniques such as group therapy, cognitive behavioral therapy, or new pharmacologic treatments in a comprehensive approach to MA-CM.

Limitations

Our study was limited by its reliance on retrospective data. The accuracy of HF and methamphetamine use diagnoses was dependent on correct documentation. It is possible that providers may have failed to diagnose either condition if they were not the primary problem. The diagnosis of methamphetamine use was supplemented by urine drug screens. Urine drug screens may have failed to capture remote cases of methamphetamine use. 

In addition, the documentation of stimulant use could not reveal the route, duration, or potency of the stimulant use. The effect of methamphetamine on the heart is believed to be mediated by cardiotoxic and sympathomimetic effects, which are proportional to the extent of exposure. It is possible that the extent of exposure could have been affected by the route, duration, or potency. Our study was also not able to quantify the rate of concomitant substance use, which has also been shown to potentiate the effect of exposure. Comorbidities were not thoroughly evaluated in this study, so any confounding comorbidities were not examined.

An additional limitation was the geographic distribution of our patients and the lack of evaluation of ethnic background. The subjects were drawn from a community hospital network with hospitals on the West Coast, South, and Midwest. These regions are not nationally representative; however, they do represent areas with particularly high concentrations of methamphetamine users [2].

## Conclusions

As methamphetamine use increases in the United States, this has prompted the development of further studies and treatment algorithms, as well as aggressive detoxification programs to reduce the rates of cardiac morbidity and mortality in this population and curve the burden on health systems across the nation. Cardiomyopathy from methamphetamine abuse is an issue, as the rate of abuse continues to rise. With current research into this subject matter, the agreed-upon therapy and treatment for MA-CM are similar to those of reduced ejection fraction cardiomyopathy, with an emphasis on patient counseling for the cessation of methamphetamine.
